# The subspecialization conundrum

**DOI:** 10.4103/0971-3026.59742

**Published:** 2010-02

**Authors:** Bhavin Jankharia

**Affiliations:** The Indian Journal of Radiology and Imaging “F”, 1^st^ Flr, Bhaveshwar Vihar, 383, Sardar V P Road, Mumbai - 400 004, India. E-mail: bhavin@jankharia.com

At a recent pediatric radiology update, a non-scientific, keypad based survey of the residents and consultants threw up some very interesting numbers.

Almost 63% said that they had received less than 2 weeks of dedicated pediatric radiology training with 82% believing that their institutions did not really place any importance to dedicated pediatric radiology training. Having said that, 45% on a scale of 1-5 believed that it was not really important to have adequate training in pediatric radiology (scores 1 and 2), whereas 23% were equivocal (score of 3), while 57% agreed that the training was inadequate as well (scores 1 and 2), 17% being equivocal (score 3).

When the questions were rephrased from a patient's perspective, 77% believed that children were receiving inadequate care with respect to medical imaging (scores 1 and 2), with another 17% were equivocal. When the question was reworded and they were asked whether the radiologists were competent or not when handling children, 47% were equivocal, 30% thought they were not competent, but 22% believed that they were competent. This is not surprising since radiologists will in general blame the system rather than themselves for deficiencies in care and will also believe that they are better than the others around them. However, surprisingly, 75% of those present thought that pediatric radiology will gain more and more importance in the future.

What this tells us in the end is that training in pediatric radiology in India is inadequate, the focus on training is all but absent and this translates into inadequate and perhaps poor care in the majority of radiology centres and departments across the country.

It is obvious that we have to subspecialize. That is the only way we will be able to speak the language of our clinical colleagues and answer the questions that they have. In India, there is some semblance of subspecialization in neuroradiology and interventional and vascular radiology, but beyond these two disciplines, there is a significant resistance to subspecialization.

So ultrasonologists who can very well afford to subspecialize in obstetrics will still do Achilles tendon scans and mammologists will also handle the testes. We often have CT scan and MRI subspecialists, but chest, cardiac, bone, gastrointestinal and genitourinary or abdominal subspecialists are few and far between.

The reasons are many; lack of training or focus during residency; a fear that organ subspecialization will lead to a reduced value in the job-market, if and when jobs have to be changed; a sense of not wanting to let go; a lack of peers around who can serve as role models, etc.

However, if we are to gain the confidence of our clinical colleagues and avoid becoming redundant, we have to start understanding that depth of knowledge is more important than breadth. Today, chest physicians can read high-resolution CT scans better than the vast majority of radiologists; neurologists and neurosurgeons are often better than the radiologists they refer scans to; most rheumatologists are far better than radiologists at reading plain radiographs, etc. Though there is a financial component involved when clinicians start doing their own imaging, there is often an equal amount of frustration at the lack of expertise around them that drives them into taking matters into their own hands.

Pediatric radiology more than any other discipline needs subspecialists. Infants and children are not just young adults. Radiologists who understand how to adjust protocols, and who can speak the same language as the pediatricians and pediatric surgeons, are sorely required, if we are to be an integral part of the teams that manage infants and children. Until then we will remain image-producers, not doctors who can make a positive difference!

